# The global challenge of advanced practice nursing (APN): a call for standardization and recognition

**DOI:** 10.15649/cuidarte.5965

**Published:** 2026-03-19

**Authors:** Lyda Z. Rojas, Angie Cristina Mendoza-Quiñonez, Liliana Andrea Mora Rico

**Affiliations:** 1 Fundación Cardiovascular de Colombia, Hospital Internacional de Colombia. Piedecuesta, Colombia. E-mail: lydarojas@fcv.org Fundación Cardiovascular de Colombia Piedecuesta Colombia lydarojas@fcv.org; 2 Fundación Cardiovascular de Colombia, Hospital Internacional de Colombia. Piedecuesta, Colombia. E-mail: angiemendoza@fcv.org Fundación Cardiovascular de Colombia Piedecuesta Colombia angiemendoza@fcv.org; 3 Fundación Cardiovascular de Colombia, Hospital Internacional de Colombia. Piedecuesta, Colombia. E-mail: lilianamora@fcv.org Fundación Cardiovascular de Colombia Piedecuesta Colombia lilianamora@fcv.org

**Expansion and definition of advanced practice nursing (APN)**

**Figure 1:**
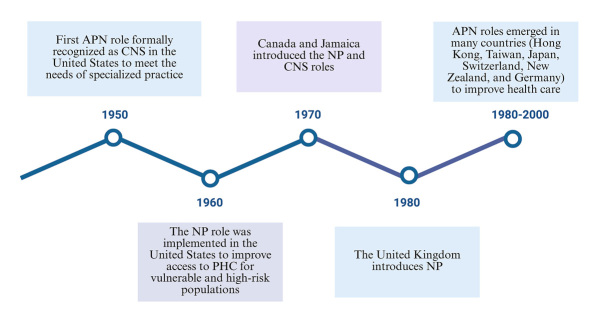
Introduction of advanced practice nursing (APN) over time

*Source: Self-developed, based on information from Almukhaini, et al.^5^. APN: Advanced practice nursing; CNS: Clinical nurse specialist; NP: Nurse practitioner; PHC: Primary health care.*

Advanced Practice Nursing (APN) originated in the United States in the 1950s as a response to emerging healthcare needs. Its inception focused on improving child health indicators in the rural and hard- to-reach areas of Colorado. To achieve this, the development of APN involved training nurses to expand their autonomy, allowing them to provide comprehensive primary health care. This included advanced competencies such as diagnosing conditions, prescribing treatments (including medications), and a focus on health prevention and promotion, surpassing the traditional competencies of nursing^1^. See Figure 1for the introduction of APN over time.

The emergence of APN is redefining the traditional delineation between medicine and nursing. Historically driven by physician shortages and the need to address the growing and changing health demands of the population, these roles are now imperative. The integration of APN is an essential strategy to increase patient access to qualified care, support existing medical staff, reduce pressure on healthcare costs, and foster continuous professional development^2^.

APN roles have matured in many countries, while others, such as France and Chile, are in the initial phases of implementation^3^. The International Council of Nurses (ICN) defines the advanced practice nurse as a «generalist or specialized nurse who has acquired, through additional postgraduate education (minimum of a master’s degree), the expert knowledge base, complex decision- making skills, and clinical competencies for Advanced Nursing Practice, the characteristics of which are shaped by the context in which they are credentialed to practice»^3^,^4^.

**The heterogeneity of APN: Titles, roles, and regulation**

According to the influential State of the World’s Nursing Report 2020, nursing plays a fundamental and indispensable role in advancing the Sustainable Development Goals (SDGs)^6^. The profession makes a critical contribution to numerous global health priorities, including achieving universal health coverage, the effective management of non-communicable diseases and mental health, strengthening patient safety, and enhancing emergency preparedness and response. Essentially, nursing ensures that the care provided is fundamentally patient-centered, serving as a driving force behind the world’s most ambitious health agendas^5^.

In response to the growing demand for health services, the World Health Organization (WHO) has advocated for expanding nursing practice to promote equity in care. Globally, these advanced roles are known by various nomenclatures, the most common being the Advanced Practice Nurse, the Nurse Practitioner (NP), the Clinical Nurse Specialist (CNS), as well as the Nurse Anesthetist and the Nurse Midwife^7^,^8^. APNs are essential because they provide direct clinical care to patients and families with acute, chronic, or complex conditions. In addition to direct care, they support healthcare teams in improving the quality of care and increasing access to services^7^. While these roles are experiencing exponential global growth, understanding their impact is hindered by inconsistencies in role definitions and titles, as well as a lack of clarity about their functions globally^7^,^9^.

It is complex to differentiate the essential functions of general practice nurses from those of Advanced Practice Nurses. This ambiguity is illustrated in jurisdictions such as Belgium, where the totality of nursing functions is grouped under the term "nursing leadership." To achieve optimal use of APN functions globally, it is essential to address this lack of clarity. A more precise understanding of the existing functions, the diverse international titles used, and the relevant outcomes associated with APN is fundamental. This will not only inform global priorities in APN education, research, and policy reform, but will also ensure that APNs’ unique impact is recognized and maximized^7^,^9^.

Although the titles, functions, and responsibilities of Advanced Practice Nurses vary significantly across countries, the benefits of implementing this role have been widely demonstrated in the clinical and economic spheres. Clinical contributions include reducing medication use in long-term care facilities, decreasing the length of hospital stay for patients with hip fractures, and lowering readmission and mortality rates in patients with heart failure. Furthermore, beyond these superior clinical outcomes, an economic impact related to the prevention of adverse events following the introduction of the APN role has also been confirmed^10^.

Regulating APN roles represents the most significant challenge to their global consolidation and expansion. The current situation is characterized by profound inconsistencies in legislation and nomenclature across different countries and jurisdictions. The lack of regulatory uniformity affects essential aspects, such as the scope of practice (what they can legally do, including diagnosing, prescribing, and ordering tests), minimum education requirements (which should be a master's degree or higher, according to the ICN), and the mechanisms for accreditation and certification. This legal and professional disparity hinders the mobility of APNs, complicates their understanding among health policymakers, and, most importantly, limits their potential to operate with the full autonomy the role requires. International and national bodies must work towards a common regulatory framework that clearly defines the competencies and responsibilities of APN to maximize its contribution to access and equity in health care^8^.

**APN in Latin America**

APN in Latin America and the Caribbean is currently in the exploratory phase of implementation as a practical resource to address challenges in accessibility (for vulnerable populations, particularly those in rural and remote communities), as well as quality and safety in care, especially within primary health care (PHC). Its full integration is hindered by persistent role confusion between Advanced Practice Nurses with master's degrees and specialist nurses with extensive experience, as well as by local legal and regulatory aspects that can limit its recognition. Therefore, advancing APN urgently requires a clear understanding of its competencies and unified nursing leadership to negotiate its implementation^11^,^12^.

Despite these challenges, several countries have initiated discussions to integrate the APN role into their models of care. Chile has emerged as a notable leader, successfully designing Master’s programs with a community/family focus and envisioning a legal framework that projects a significant improvement in the resolution capacity of PHC; this progress allows APNs to manage patients with chronic conditions and modify treatments in home care. For its part, Colombia is among the countries that have begun these discussions, recognizing the urgency of establishing specific legislation for APN, not only as a professional debate but as a public health necessity to reduce morbidity and mortality and expand the profession’s prestige and salary recognition^13^. In parallel, Brazil stands out for its robust infrastructure of postgraduate programs, meeting the conditions to implement the Nurse Practitioner (NP) role with a PHC focus, which is key to improving population health. Nevertheless, implementing education in Brazil requires adapting it to the ICN recommendations and overcoming the challenge of regulating its scope of practice. Overall, the regional challenge requires nursing schools to lead curricular review processes and foster a shared identity to standardize the future of the role^14^,^15^.

In conclusion, the exponential growth of APN worldwide, along with its undeniable positive impact, is indisputable. Nevertheless, the global inconsistency in definitions, titles, and regulatory frameworks hinders the full realization of its potential. To ensure that APN achieves its maximum value, a determined and coordinated commitment from all stakeholders—educators, regulators, and legislators—is essential to standardize education, clarify competencies, and formalize role regulation within its own theoretical framework. Only in this way will it be guaranteed that APN is recognized and optimally utilized in the global strategy to achieve universal health.
